# Comparing mapped park and greenspace boundaries in Philadelphia: implications for exposure assessment in health studies

**DOI:** 10.1186/s12942-024-00370-x

**Published:** 2024-08-31

**Authors:** Dustin Fry, Lara A. Roman, Michelle C. Kondo

**Affiliations:** 1 USDA Forest Service Northern Research Station, 100 North 20th Street #405, Philadelphia, PA 19103 USA; 2 USDA Forest Service Northern Research Station & Pacific Southwest Research Station, 4955 Canyon Crest Drive, Riverside, CA 92507 USA

## Abstract

An important consideration in studies of the relationship between greenspace exposure and health is the use of mapped data to assign geographic exposures to participants. Previous studies have used validated data from municipal park departments to describe the boundaries of public greenspaces. However, this approach assumes that these data accurately describe park boundaries, that formal parks fully capture the park and greenspace exposure of residents, and (for studies that use personal GPS traces to assign participant exposures) that time spent within these boundaries represents time spent in greenspace. These assumptions are tested using a comparison and ground-truthing of four sources of mapped park and greenspace data in Philadelphia, Pennsylvania: PAD-US-AR, Philadelphia Parks and Recreation, the Delaware Valley Regional Planning Commission, and Open Street Maps. We find several important differences and tradeoffs in these data: the incorporation of highways and building lots within park boundaries, the inclusion or exclusion of formal park spaces (federal, state, and nonprofit), the exclusion of informal parks and greenspaces, and inconsistent boundaries for a linear park. Health researchers may wish to consider these issues when conducting studies using boundary data to assign park exposure.

## Introduction

Questions regarding whether and how urban parks and greenspace affect health have received increasing attention in public health literature [1]. Studies have found beneficial associations between living near parks and visiting parks on both physical [2] and mental [3] health outcomes.

An important consideration in research on the effect of parks on health is exposure assessment. Many studies have used self-reported measures to assign exposure, such as participants’ perceived access to [4], or reported use of, parks [5]. Alternately, common objective measures include distance from participant residences to the nearest park [6] or the total area of parks [7] or greenspace [8] within a certain distance of participant residences. These objective and subjective measures of park access do not always correspond [9, 10], and differences between the two can be associated with other aspects of the built and social environment [11, 12].

Widespread availability of personal GPS traces from cell phone data [13], personal monitoring devices [14], or from online platforms [15], provide an opportunity for park exposure assessment to be based on participants’ objectively-measured locations with high spatial and temporal fidelity. However, the validity of this method depends both on the accuracy of the GPS traces and on the accuracy of the mapped data that is used to determine the location of park boundaries. Inaccuracy in park location data can lead to misclassification of proximity or determination of whether a participant is inside a park at a given time. Inaccurate park boundaries could result from excluded areas outside of jurisdictional boundaries or organizational interests, infrequent updates, or lack of classification or exclusion of non-park areas. For example, park data may not appropriately deal with intersecting roadways, which could be experienced as separate to a park or a feature of it.

In addition, greenspace exposure estimates may not account for administrative park classifications. Public parks often include not only outdoor vegetated areas, but also paved areas and indoor facilities such as recreational centers, administrative buildings, and museums [16]. While these facilities can be beneficial in promoting exercise [17, 18], socialization [19], and cooling [20], they are not greenspace. Conversely, participants can be exposed to greenspace in areas other than public parks, such as residential lots [21], greened vacant lots [22], community gardens [23], and private environmental centers [16], arboreta, or botanical gardens [24]. Even if these spaces are publicly accessible, they may not be included in readily-available mapped data. These concerns limit researchers’ ability to use park visitation, park access, or park proximity as a proxy for greenspace exposure.

Any of the above issues in isolation or combination could result in exposure misclassification. If the accuracy of mapped data varies across a study area, differential misclassification could pose a threat to study validity, meaning that errors in exposure assessment could also be associated with outcomes and result in substantial bias. Alternately, if the total park coverage from each data source is different but the geographic distribution of park coverage is the same, differential misclassification may be less of a concern. If researchers are aware of the strengths and weaknesses of the data at their disposal, they can select data that best mitigates against bias in exposure classification [25].

Given the research challenges with assessing park exposure, our aim was to examine the limitations of publicly-available data sources for the purpose of providing individual greenspace exposure estimates in GPS-based studies by comparing four sources of park/greenspace boundary data within the city limits of Philadelphia, Pennsylvania, USA. We made the following comparisons of the data sources: (1) the overall agreement of each dataset; (2) the proportion of parkland/greenspace in each planning district; (3) the extent to which informal public greenspaces are included; and (4) fine-scale qualitative assessments and ground-truthing of several selected areas in Philadelphia.

## Methods

### Study setting

The study setting is the City of Philadelphia (population 1.6 million in 2023) in the northeast region of the USA. Built and social environmental characteristics vary widely across the city; population density ranges from less than 500 residents/km^2^ to over 8,500 residents/km^2^. Philadelphia is one of the most racially segregated cities in the United States [26], and historically redlined Philadelphia neighborhoods tend to have lower levels of tree canopy in the present day [27]. We center our analysis on parks and greenspaces in Philadelphia, including a variety of outdoor vegetated landscapes that are free to the public, as well as some greenspaces that are open to the public but have admission fees, and others with restricted access.

The municipal Parks and Recreation department (PPR) manages 40 square kilometers of public parkland within Philadelphia [28], including large watershed parks and smaller neighborhood parks and recreation centers. Philadelphia is also home to substantial national and state-managed public parkland including the John Heinz National Wildlife Refuge (Fish and Wildlife Service), Independence National Historical Park (National Park Service), and Benjamin Rush State Park (Philadelphia Department of Conservation and Natural Resources).

Private institutions and non-profit organizations manage additional parks and greenspaces including environmental centers (e.g., Schuylkill Center for Environmental Education), arboreta (e.g., Morris Arboretum and Gardens of the University of Pennsylvania), and cemeteries that serve important park-like functions [29]. There are also small neighborhood parks and open spaces on university and corporate campuses, as well as vacant lots and community gardens with varying levels of formal maintenance. The non-profit Pennsylvania Horticulture Society holds the primary contract to “clean, green, and maintain” vacant lots for public benefit across the city through its LandCare program, formalizing and facilitating a process to increase residents’ access to public greenspaces [30]. These various parks and open spaces that are not administered by government departments may be accessible to the public free of charge or with paid admissions, or inaccessible to the public and restricted to certain people (such as university ID holders).

### Data sources

We selected four publicly available data sources providing boundaries for park and greenspace areas:

1. the Philadelphia Parks and Recreation Department (PPR) Properties map. PPR maintains a spatial database of properties, including parks and recreational facilities, for which PPR has a role in maintenance or management. The data is updated weekly and is available for download at OpenDataPhilly [28].
2. the Delaware Valley Regional Planning Commission (DVRPC) Protected Open Space Inventory map. The inventory “tracks all publicly-owned open space, preserved farmland, and non-profit protected open space” within an eight-county region centered on Philadelphia [31]. The inventory is updated every four years and is available for download at DVRPC’s ArcGIS Online site [32].
3. park and greenspace data from Open Street Maps (OSM). OSM is a global open-source mapping project that is continuously maintained and updated by volunteers and available for download [33]. We assembled the OSM map by merging the OSM Natural Areas layer with all features in the Land Use layer with “type” of either nature_reserve, park, cemetery, forest, recreation_ground, allotments, scrub, meadow, grass, or heath, and all features in the Points of Interest layer with “type” of either park, graveyard, golf_course, picnic_site, or pitch.
4. the PAD-US-AR map. PAD-US-AR is a curated version of the USGS Protected Areas Database of the United States that identifies protected areas that are accessible to the public for outdoor recreation [34]. The researchers responsible for the creation of PAD-US-AR have made the map available for public download [35]. Unlike the other three data sources, which are curated at a local level, the PAD-US-AR is intended for use in national- or regional-scale analyses [34]. Nonetheless, the boundaries provided in this data source display individual outdoor recreation parcels within cities, leaving open the possibility for its use where other sources of data are not available.

The first two sources are specific to the Philadelphia area while the latter two are available nationwide. We clipped all maps or spatial datasets to the Philadelphia city limits.

### Overall agreement

Overall agreement was assessed by overlapping the park boundaries provided in all data sources. Agreement is reflected by the areas enclosed by multiple data sources. Agreement was also computed for each individual data source, comparing the overlap of its boundaries with the rest of the data sources.

*Comparison of Parkland/Greenspace by Planning District.* Although we lack a gold standard as to what the “true” distribution of parks and greenspaces is within the city, differences in the way that estimated park/greenspace coverage is distributed across the city by data source could also imply departures from actual conditions and therefore reflect a risk of differential misclassification across space. For the purposes of city planning, 18 planning districts are administratively defined for the City of Philadelphia, which roughly correspond with groups of commonly-understood neighborhoods [36]. To assess whether overall estimates of park and greenspace area differ across the city, we computed the ratio of park/greenspace to total area (including land and water areas) for each planning district. For each planning district, we computed the coefficient of variation between data sources. Larger coefficients of variation reflect greater proportional differences in park and greenspace estimates.

### Inclusion of informal greenspaces

Philadelphia has large numbers of small and/or informally managed greenspaces including pocket parks, community gardens, and greened vacant lots. Because these types of parks and greenspaces might not be under the purview of government agencies, recreation departments, or other large organizations, these greenspaces can be difficult to characterize in readily-available data. However, an available source of mapped data for greened vacant lots managed under the LandCare Program may be used as a proxy to understand the inclusion of these lots in other data sources. Although LandCare lots do not represent the full set of greened vacant lots or other informal parkland or greenspaces, their relative inclusion or exclusion from other mapped data may signal broader patterns of each data source’s likelihood of including such spaces.

We obtained a map of all LandCare greened vacant lots current as of March 2023 from Open Data Philly [37]. We then classified each lot based on whether its center is located within a feature of the mapped data under analysis: PPR, OSM, PAD-US-AR, and/or DVRPC. Greater overlap between the LandCare lots and the other data sources suggests greater inclusion of informal green spaces, while low overlap suggests exclusion of informal green spaces.

### Qualitative analysis

To assess specific differences between data sources, we conducted a qualitative analysis of selected areas of Philadelphia. This included visual comparisons of the four data sources as well as photographic ground truthing of these areas. We selected West Fairmount Park, Lower Southwest Philadelphia, Independence Mall, University City, and Northeast Philadelphia as areas that are likely to demonstrate divergence between the different maps because of the locations of parks under management by different public and private organizations, multiple roads and highways that pass through park and greenspace areas, and the presence of parks containing areas of both greenspace and buildings. Finally, we included the Schuylkill River Trail (SRT) as an important linear park. The SRT presents a complex mapping problem because along its 15-kilometer length within Philadelphia it intersects many roads (both at grade and grade separated) and it has undergone multiple extensions and course changes over the past decade.

## Results

### Overall agreement

**Map. 1:**
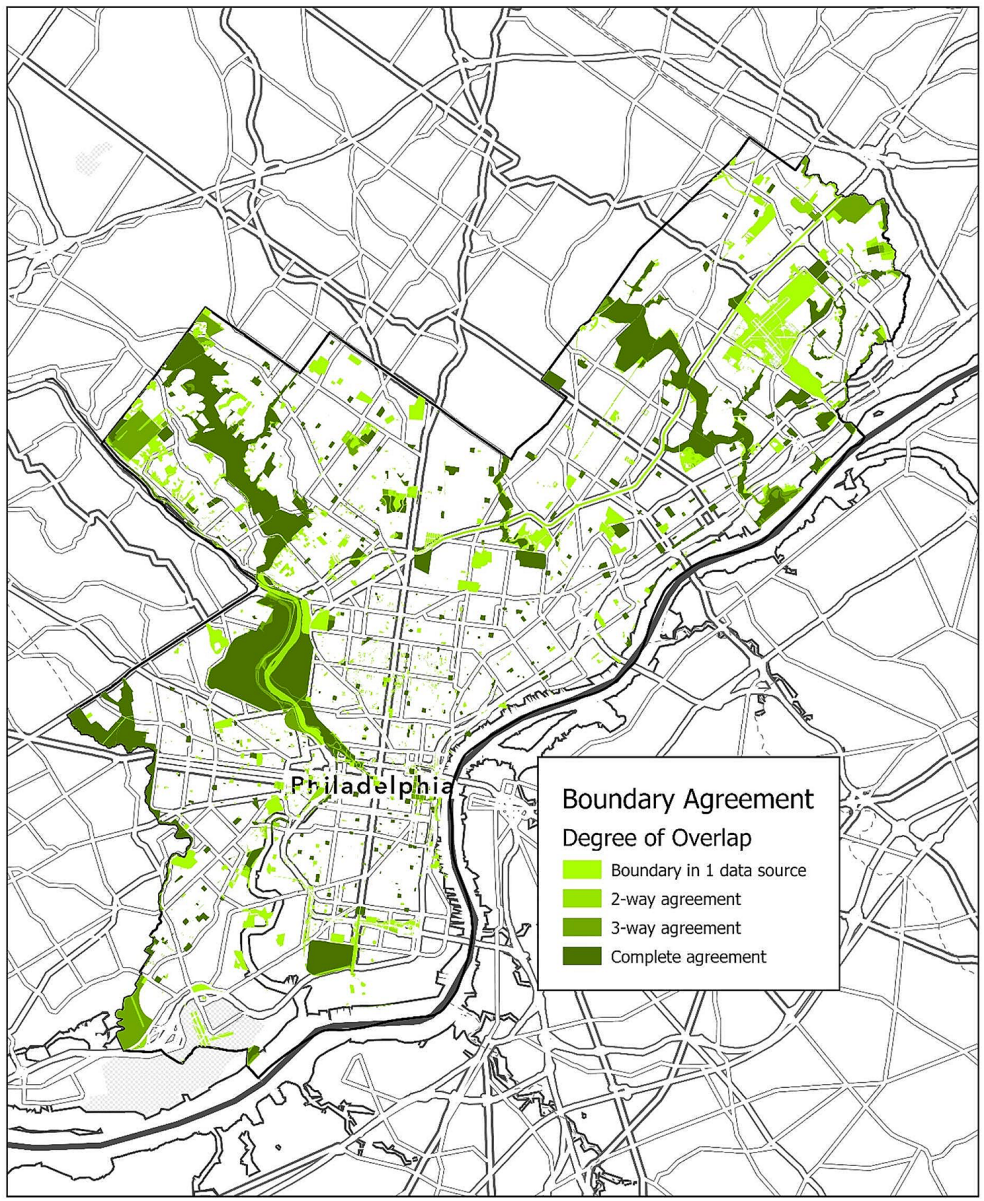
Overall agreement between data sources. Map. 5 symbolizes the overall agerement between data sources by showing the overlap of park and greenspace boundaries across the city of Philadelphia. Darker features are represented in more data sources than are lighter features, with the darkest color symbolizing overlap of all data sources and the lightest color symbolizing that only one data source includes that feature

Table 1 displays overall levels of agreement between all data sources and for each individual data source. 50.1% of the total area enclosed by any data source’s park boundaries is enclosed by all data sources, while 33.0% of the total enclosed area is represented only in one data source. The PPR boundaries have the lowest total area (39.86 km) and the greatest level of agreement with the other data sources, with 88.8% of the PPR’s area enclosed by all other data sources. OSM has the highest total area and the lowest level of agreement, with 30% of its total area not being shared by any other data source’s boundaries. This result is also displayed in Map 1.

**Table 1:** Rows display the number of data sources providing overlapping boundaries. “No overlap” indicates areas that are enclosed by boundaries in only one data source, while “complete agreement” indicates areas that are enclosed by the boundaries of all four data sources. 2-way agreement and 3-way agreement indicates areas that are enclosed by any two or any three data sources, respectively. Data-specific columns show agreement between each individual data source and the rest of the group

Number of Overlapping Boundaries	Overall		OSM		PPR		DVRPC		PADUS-AR	
	Area (km^2^)	% of Total	Area (km^2^)	% of Total	Area (km^2^)	% of Total	Area (km^2^)	% of Total	Area (km^2^)	% of Total
No Overlap	23.35	33.0%	18.87	30.0%	0.40	1.0%	0.35	0.8%	3.73	7.5%
2-way agreement	4.16	5.9%	3.09	4.9%	0.98	2.5%	1.19	2.7%	3.07	6.2%
3-way agreement	7.82	11.1%	5.58	8.9%	3.06	7.7%	7.59	17.0%	7.24	14.6%
Complete agreement	35.41	50.1%	35.41	56.3%	35.41	88.8%	35.41	79.5%	35.41	71.6%
Total	70.75	100%	62.95	100%	39.86	100%	44.54	100%	49.45	100%

### Comparison of parkland/greenspace by planning district

**Map. 2:**
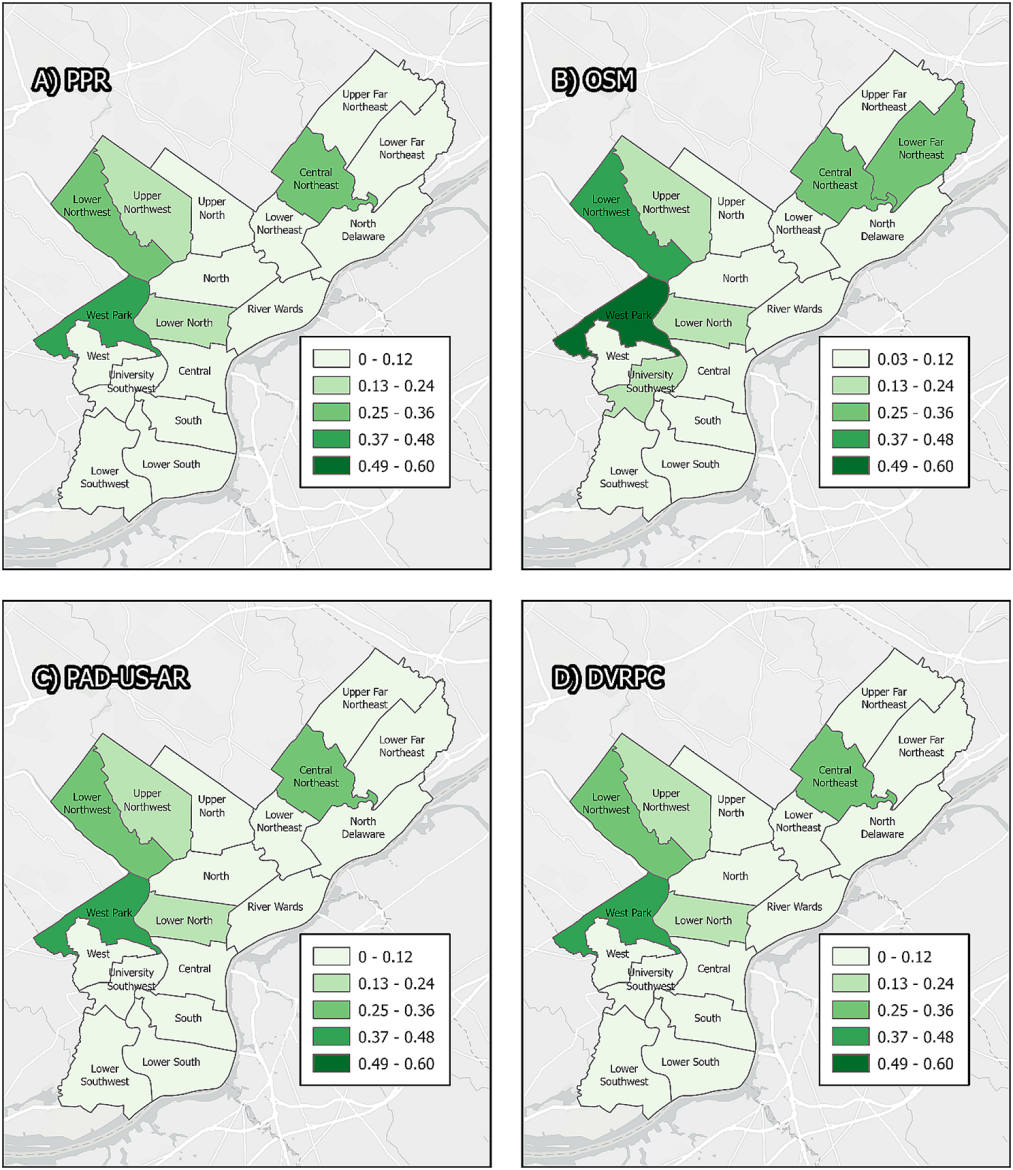
Philadephia planning districts. Panels display Philadelphia planning districts, symbolized by the proportion of area of each district that is categorized as greenspace or parkland by four different sources of data: the Philadelphia Parks and Recreation Department, Open Street Maps, PAD-US-AR, and the Delaware Valley Regional Planning Commission. Individual districts may fall into different categories of park/greenspace density depending on the source of data used, because different data sources delineate different boundaries and have different eligible properties. For example, the Lower southwest contains the John Heinz National Wildlife Refuge, which is included in all sources of data except PPR

For each of 18 planning districts in Philadelphia, Table 2 displays the proportion of park/greenspace as calculated from each source of assessed data, as well as the overall mean and coefficient of variation. The table shows reasonable agreement between the data sources at the district level for most districts. For some districts, such as the Lower Far Northeast, University Southwest, and Lower Southwest, there are large discrepancies. Depending on the neighborhood of residence for a study participant, different data sources could provide considerably different area-level exposure estimates for parks or greenspace. This result is also displayed in Map 2.

**Table 2:** Proportion of parks or greenspace in each planning district by data source

District	PPR	OSM	PAD-US-AR	DVRPC	Mean	CV
Lower Far Northeast	0.100	0.248	0.114	0.095	0.139	0.523
University Southwest	0.056	0.147	0.080	0.054	0.084	0.514
Lower Southwest	0.024	0.113	0.076	0.077	0.073	0.504
Upper Far Northeast	0.028	0.112	0.084	0.066	0.073	0.487
River Wards	0.014	0.030	0.015	0.014	0.018	0.429
North	0.056	0.120	0.061	0.056	0.073	0.428
Upper North	0.053	0.111	0.071	0.050	0.071	0.392
Lower Northeast	0.056	0.118	0.077	0.056	0.077	0.381
South	0.029	0.057	0.034	0.031	0.038	0.348
Lower South	0.062	0.096	0.069	0.062	0.072	0.222
Central	0.070	0.101	0.106	0.077	0.088	0.200
Lower Northwest	0.248	0.388	0.307	0.301	0.311	0.187
Lower North	0.156	0.219	0.180	0.156	0.178	0.168
West	0.046	0.057	0.046	0.044	0.048	0.125
Upper Northwest	0.150	0.197	0.187	0.173	0.177	0.115
North Delaware	0.096	0.109	0.119	0.098	0.106	0.099
West Park	0.412	0.483	0.461	0.442	0.449	0.066
Central Northeast	0.276	0.288	0.285	0.272	0.280	0.027

### Inclusion of informal greenspaces

A total of 9,663 greened vacant lots were identified as part of the LandCare Program. Of these, 8 lots were within a PPR property; 1,914 within an OSM park or greenspace; 7 within a DVRPC protected open space; and 15 within a PAD-US-AR greenspace. At points of intersection, these maps tended to reflect the same LandCare lots: 7 lots had their center in all four sources of data.

Overall, a small proportion of greened vacant lots were included in any of the park data, with Open Street Maps including the largest number. Despite being an important source of greenspace for Philadelphia residents, greened vacant lots are not represented except in specialized maps.

### Qualitative analysis

Visual comparisons of the maps in selected areas of Philadelphia provided insights into the similarities and differences in how each data source addressed jurisdictional boundaries, intersecting roads or highways, and buildings. We also evaluated the mapping of the Schuylkill River Trail with attention to the completeness of its representation.

### Jurisdictional concerns

Philadelphia parks and greenspace are managed by a diverse group of organizations, including national, state, and local parks as well as private and informal parks. Organizations publishing maps of parks and greenspace in Philadelphia may limit their maps to certain kinds of parks. With the exception of the PPR Properties map, the data assessed here include municipal, state, and national parks (such as the John Heinz National Wildlife Refuge) but all maps vary in their inclusion of private parks. Both OSM and PAD-US-AR include private greenspace managed by the University of Pennsylvania and Drexel University in the University City area of Philadelphia, but the exact boundaries vary, and the DVRPC and PPR maps exclude them entirely. Only OSM includes graveyards and cemeteries, some of which have initiatives to increase outdoor recreational use [38, 39].

### Inclusion of road and highway areas

**Map. 3:**
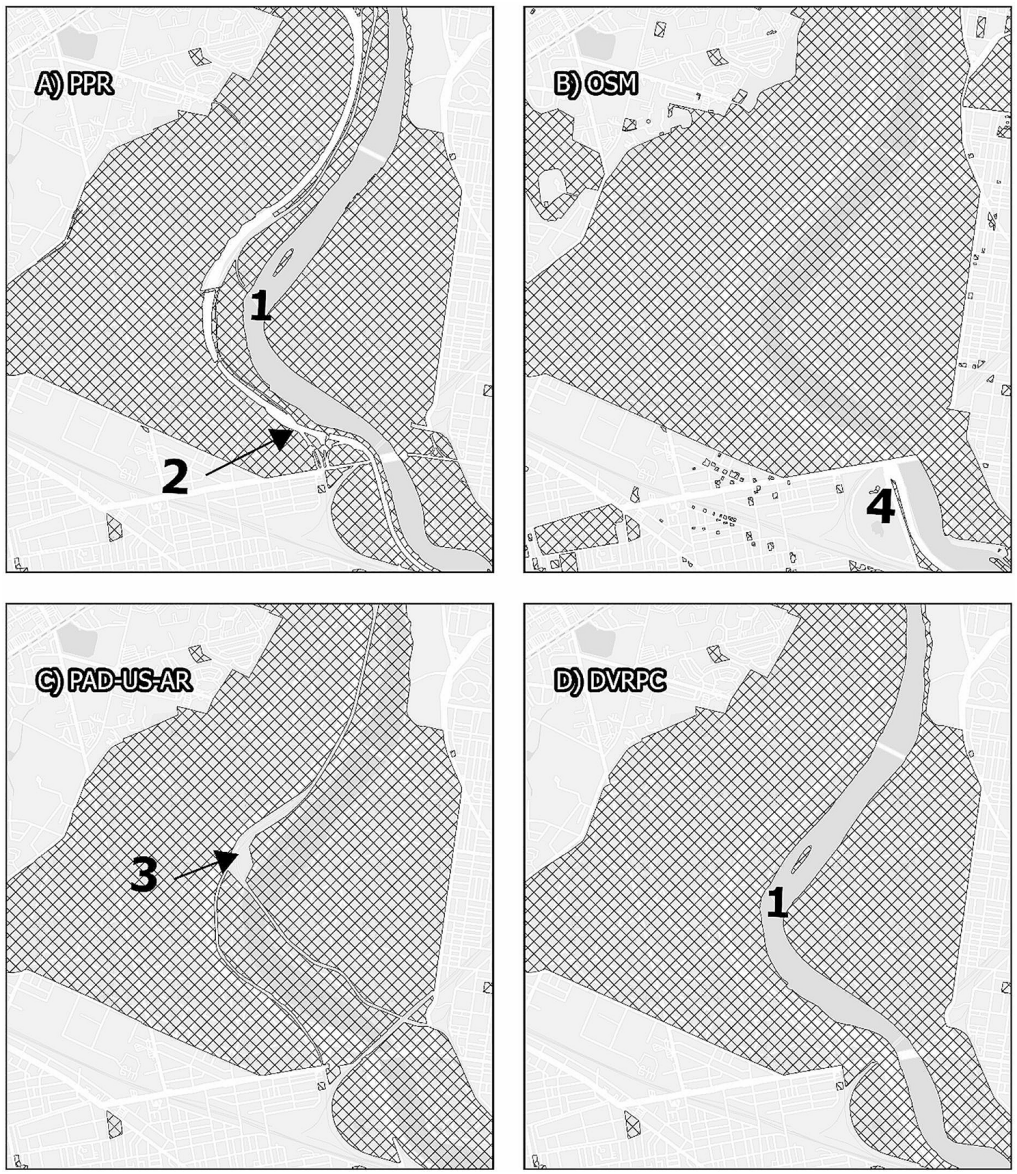
Fairmount Park. Panels display the area surrounding Fairmount Park, comparing map data from the Philadelphia Parks and Recreation Department, Open Street Maps, PAD-US-AR, and the Delaware Vally Regional Planning Commission. While all maps display similar boundaries for Fairmount Park itself, smaller park areas within the larger park are treated differently. While PPR and DVRPC exclude the Schuylkill River from the boundaries of the park (1), Open Street Maps and PAD-US-AR includes the river. Only PPR excludes Interstate 76 from the park features (2), including as parkland only pedestrian-accessible underpasses. Only PAD-US-AR excludes rain infrastructure from the park features (3). No data excludes local roads from park features, although this area includes large and heavily-trafficked roadways such as Belmont and Montgomery Avenues to the west and Kelley Drive to the east. All maps other than OSM include the Philadelphia Zoo (4)

**Map. 4:**
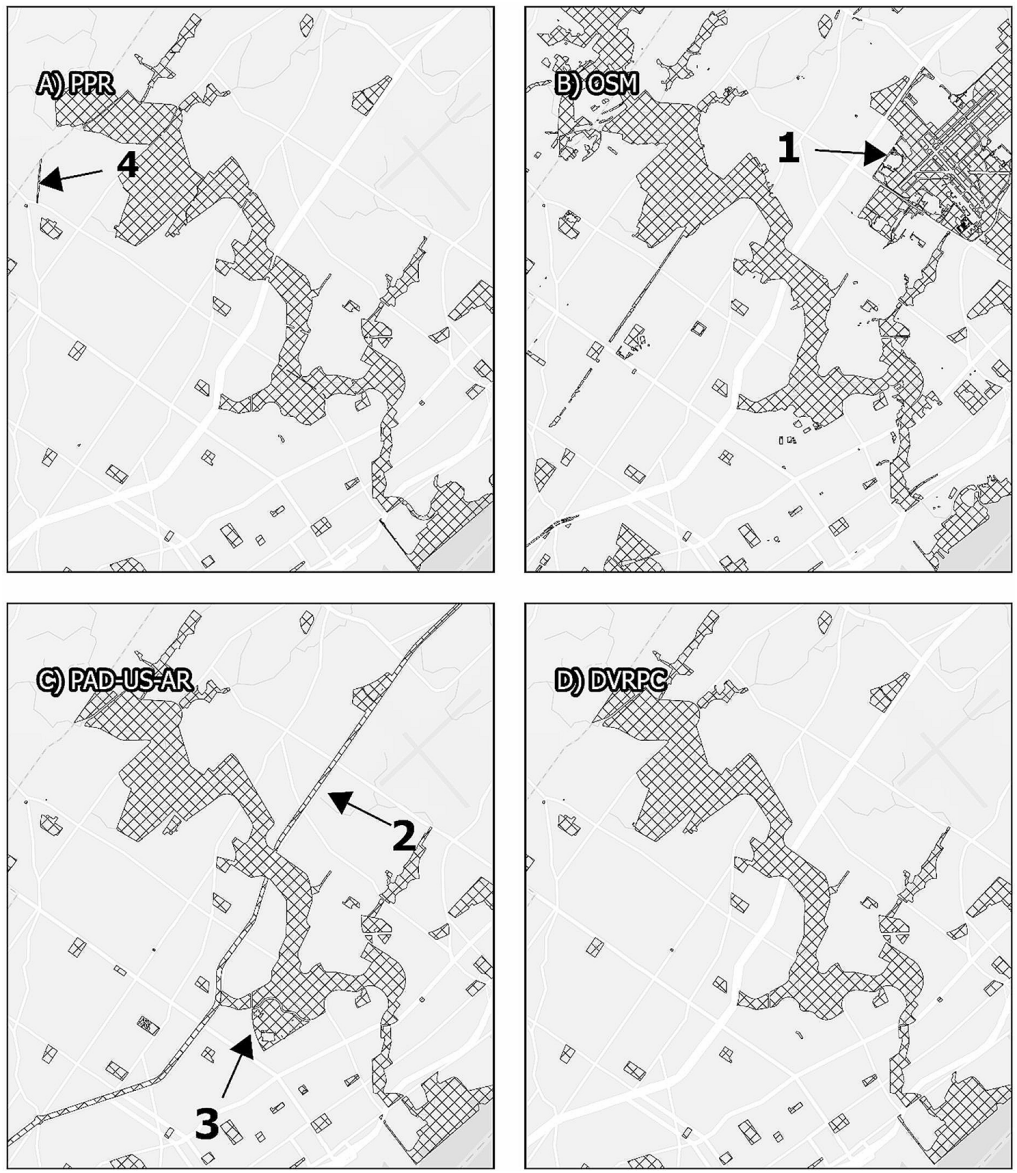
Pennypack Park and surroundings. Panels display the area surrounding Pennypack Philadelphia, comparing map data from the Philadelphia Parks and Recreation Department, Open Street Maps, PAD-US-AR, and the Delaware Vally Regional Planning Commission. While all maps display similar boundaries for Pennypack Park itself, Open Street Maps includes fields between runways of the Northeast Philadelphia Airport as grassland (1), and PAD-US-AR operationalizes the median of an urban highway (2). PAD-US-AR is also the only data source that includes the entirety of the Abraham Lincoln High School campus as a park (3). PPR is the only data source to include the Lorimer Trail Greenway (4)

**Fig. 1:**
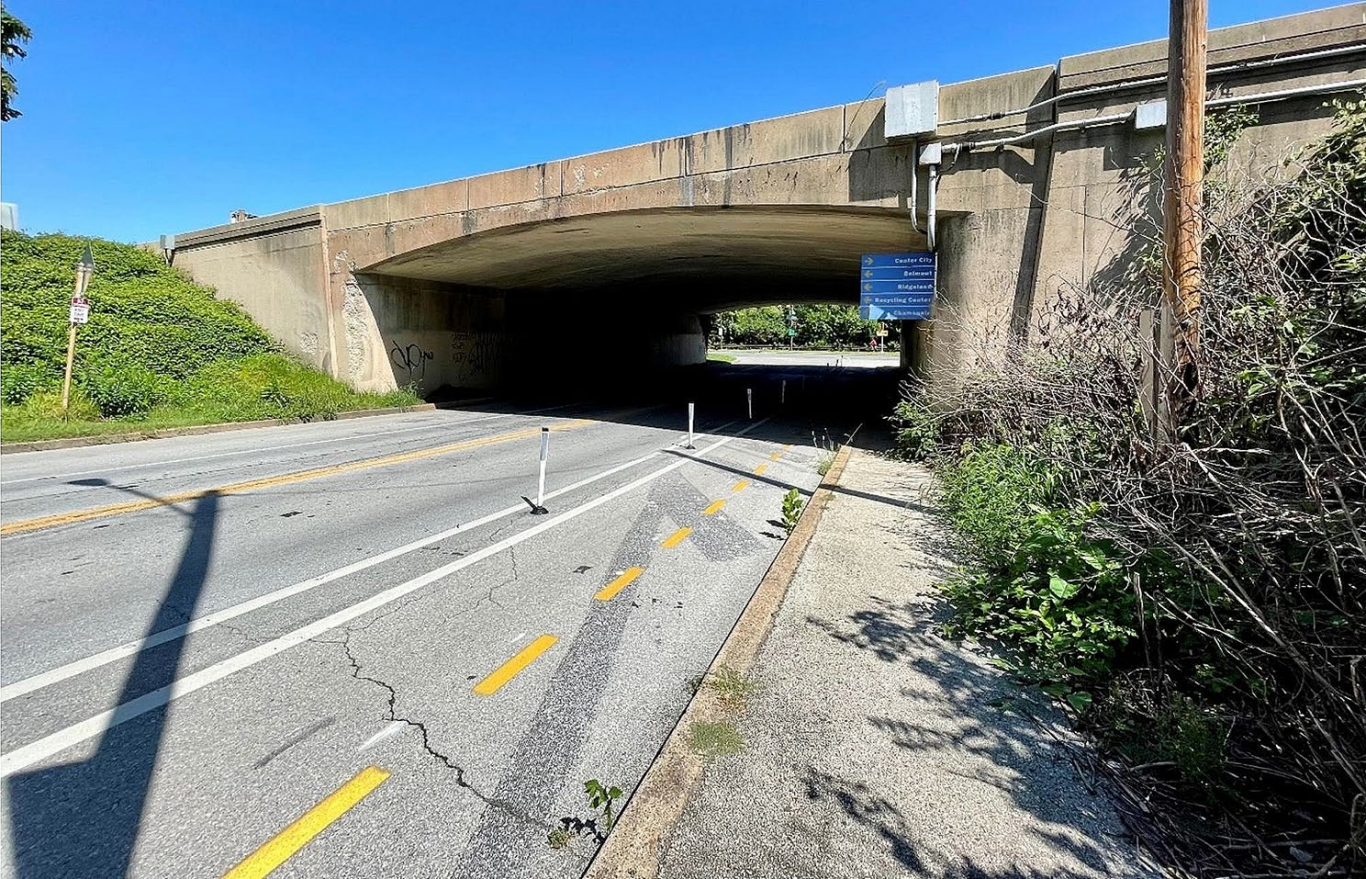
Sweetbrair Drive provides a pedestrian-accessible path underneath I-76 in Fairmount Park and is included in the PPR map

**Map. 5:**
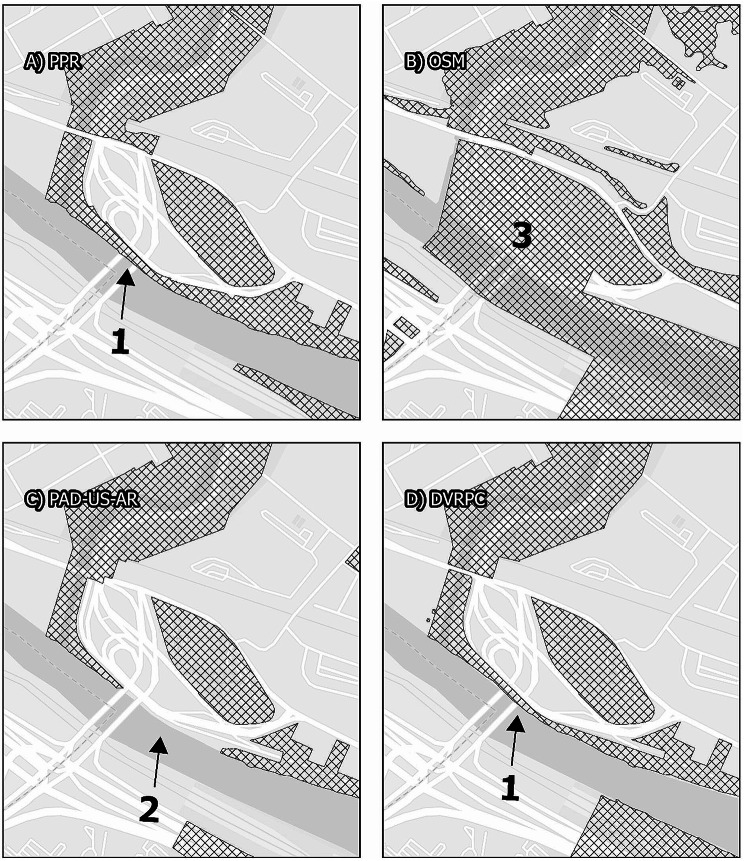
Schuylkill River Trail. The Schuylkill River Trail is a linear park that forms a substantial component of Philadelphia’s park infrastructure, and that in some places proves difficult to represent in mapped data. These panels display the area where the Schuylkill River Trail, alone the northeastern bank of the Schuylkill River, passes underneath US-1. In data from the Philadelphia Parks and Recreation Department and the Delaware Valley Regional Planning commission, this is represented as a thin feature along the riverbank that intersects the highway (1). In contract, PAD-US-AR excludes the entire highway interchange (2), despite the fact that there is parkland underneath it, and Open Street Maps includes the entire area as a continuous feature (3), although some areas are inaccessible due to the highway infrastructure

Each data source included highway and road crossings in different ways. As illustrated in Maps 3, 4, 5, local roads that bisect parks are included within park boundaries for all data sources. However, intersections with interstate highways are represented differently. Fairmount Park is a large park that covers both the east and west sides of the Schuylkill River. For example, Interstate 76, a major north-south federal controlled-access highway, runs parallel to the river, with a small buffer between the highway and the river that incorporates a local roadway, a mixed-use trail with points of river access, and some riparian vegetation. Map 3 illustrates that only the PPR map excludes the majority of Interstate 76 from Fairmount Park, though it does include accessible pedestrian underpasses (Fig. 1). This means that GPS traces following roadways that intersect large parks, for example from Interstate highway drivers, could be categorized as park visitation.

**Fig. 2:**
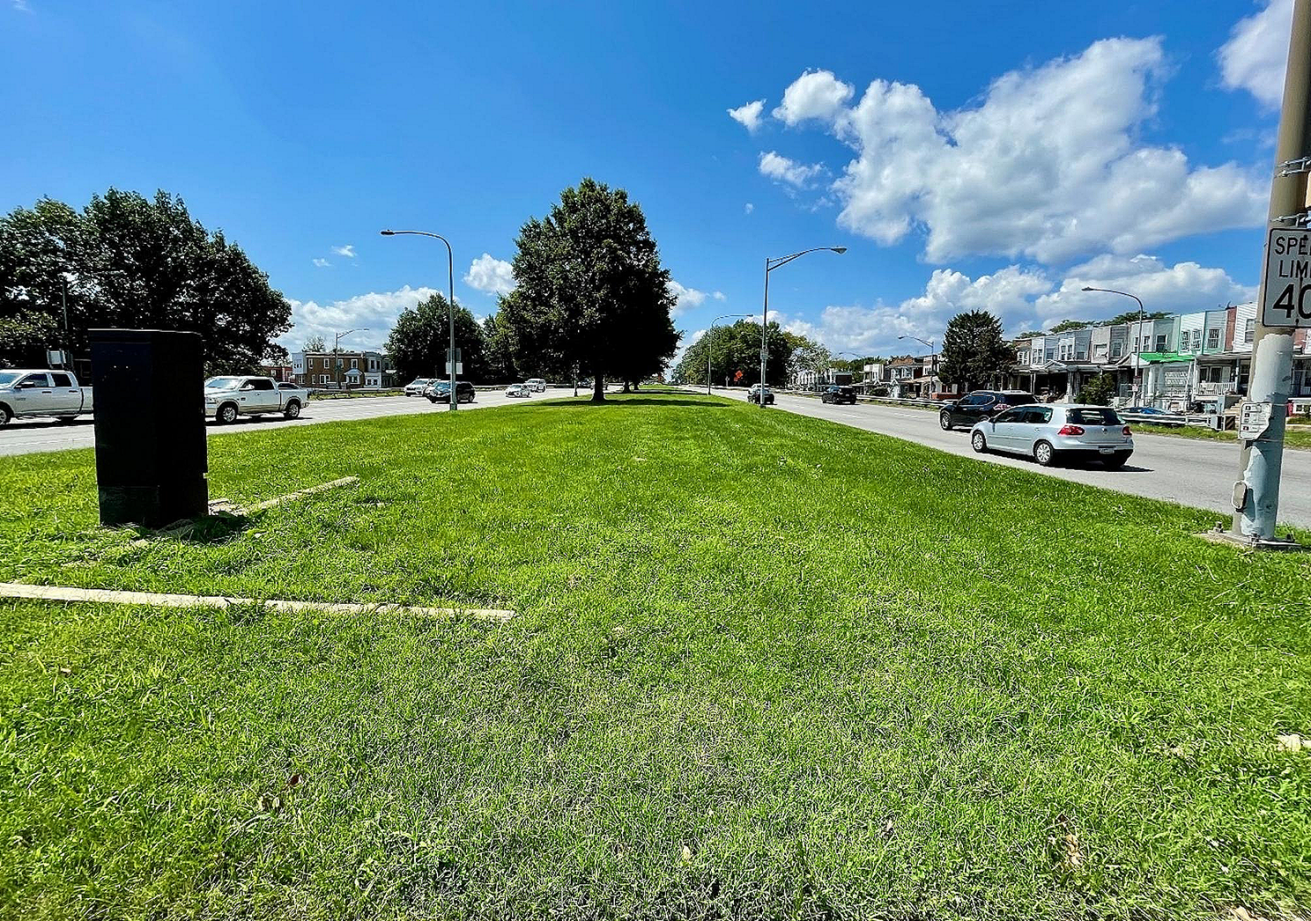
Roosevelt Boulevard is included in the PAD-US-AR map

Similarly, Map 3 illustrates that PAD-US-AR categorizes the entirety of a surface-level federal highway (Roosevelt Boulevard/US Route 1) as parkland. This Boulevard has grassy or tree-lined medians for much of its length that are accessible via pedestrian crossings. However, in other areas, the median consists of only a narrow strip of grass that is made largely inaccessible by heavy motor vehicle traffic and limited pedestrian crossings (Fig. 2), and the PAD-US-AR map includes both the medians and the roadway itself within the park area.

Another major road in Philadelphia, the Benjamin Franklin Parkway, is included differentially between data sources. The Parkway includes medians with sidewalks and tree canopy, as well as enclosing the multi-use public open space Eakins Oval. In addition, the Parkway is periodically closed to motor vehicle traffic to accommodate local events such as bike rides and running races [40]. At different times, both the roadway and its medians can function effectively as public parkland. Both OSM and PAD-US-AR include the entirety of the Parkway as a park feature, while PPR and DVRPC include only the medians and Eakins Oval.

GPS traces that follow roads or highways through a park could be classified as either exposed or not exposed depending on the data source and the road in question.

### Inclusion of buildings

While publicly-accessible buildings can have functions that are similar to parks, such as facilitating social contacts, education, recreation, and exercise, researchers may wish to isolate exposure to outdoor park spaces. Park boundaries include building footprints in all data sources. The boundaries of Fairmount Park in Map 3incorporate multiple buildings, including a children’s museum and several historic buildings and homes. Independence Mall is a national historical park with multiple buildings and museums; these are included in park boundaries among all mapped data except PPR, which does not manage this site.

### Schuylkill River Trail (SRT)

Urban greenways are often important greenspaces for cities because they can support recreational uses as well as walking and cycling for transportation [41]. None of the data sources fully cover the SRT. Each map excludes the length of the SRT north of Shawmont Avenue. Further, the maps differ at intersections. Map 5illustrates the location where the SRT passes underneath a major highway interchange at City Avenue. Each data source chooses a different pattern of exclusion and inclusion. While users of the SRT pass safely underneath the highway overpass, separated from road traffic in the vertical dimension, this proves difficult to fully map in two dimensions. PAD-US-AR inappropriately excludes all intersecting highway areas and OSM inappropriately includes all highway areas even where there is no park or greenspace underneath (See Map 5).

## Discussion

This comparative analysis highlights several limitations of mapped park data for use in health studies, particularly studies using personal GPS traces. First, due to reasons of jurisdiction, organizational interest, or data availability, none of the assessed data sources provide universal coverage of greenspaces in Philadelphia that are readily available for visitation and use by the general public. Second, all data sources include non-park areas, including highly trafficked local roads and Interstate highways (for all data other than PPR). These non-park areas are frequently used by people who are not visiting parks and interrupt the movement of park users within park areas. Third, all data sources include buildings within park areas. Fourth, the complexity of an important linear park (the Schuylkill River Trail) is represented inconsistently between data sources.

Study participants’ access to greenspace is based on their own personal habits and patterns of movement, and individuals may pass through multiple governmental and organizational jurisdictions throughout the course of a day. Data that reflects single organizational or administrative perspectives may lack relevance to daily lived experiences and may underestimate time spent in parks and green space. Depending on the patterns of movement of an individual participant, all data sources have the potential to either under- or over-estimate park attendance and greenspace exposure because they both include non-green areas and exclude accessible green areas. These inclusions and exclusions differ between planning districts between the data. No data source that we assessed would appear to be a seamless choice for use in health studies, and therefore researchers may wish to edit existing data sources before use in analysis rather than rely on previously-validated mapped data. Researchers without their own locally-situated knowledge of a city may also need to consult local experts, such as planning departments, park staff, or community organizations. Park and greenspace boundary data could also be supplemented with other kinds of data, such as the normalized difference vegetation index (NDVI) from remote sensing [42].

Our analysis is limited by its scope. We have compared four existing data sources, and although we have discovered differences, we are not aware of a “gold standard” for parks and greenspace. Although we have ground-truthed several specific areas in Philadelphia using our local knowledge about urban park and greenspace context and complexities, examining all data sources at all locations is beyond the scope of this analysis. Therefore, in general, we are limited in our ability to make specific statements about the accuracy of each data source. In addition, while all data sources other than PPR cover areas outside of Philadelphia, our analysis was focused on Philadelphia and we cannot assume that our findings are generalizable outside of the city.

Studies that use GPS traces to assign park and greenspace exposure have taken various approaches to avoid some of the limitations highlighted here. Some studies focus on attendance patterns of specific parks [43]. By using selected parks with known boundaries as the unit of analysis, misclassification based on park definitions is not relevant, but a park-based unit of analysis cannot directly link levels of park attendance with individual-level health outcomes. Some studies seek to use robustly validated mapped data for park boundaries, excluding from consideration areas that cannot be positively affirmed as parks [13]. Although this strategy reduces the risk of including non-park areas, it increases the risk that relevant public greenspaces are excluded from analysis (i.e., increasing specificity at the cost of sensitivity).

Given the multiple options often available, researchers seeking to use mapped park data to link participant GPS traces with parks and greenspaces can carefully consider their choice of data during both study planning and interpretation. If the data used for a study matches the researchers’ hypothesis for how the park or greenspace exposure affects health, then its inclusions and exclusions will not compromise validity. Our analyses of overall agreement and of the inclusion of informal park spaces demonstrated a tradeoff between inclusion and agreement. Overall, about 50% of the total greenspace area measured by all mapped data sources is shared by all data sources. OSM, which included the most overall greenspace and the highest number of LandCare lots, has the lowest level of agreement with other data sources. Conversely, the jurisdictionally-limited PPR map has the lowest level of total parks and greenspace, but nearly 90% of the space it does include is validated through its inclusion in other maps. Restricting analyses only to those most reliable and validated park and greenspace boundaries may risk removing important greenspaces that are less likely to be captured in official park maps.

Researchers may wish to carefully consider what kinds of entries into park or greenspace areas count as visitation under their theoretical framework and ensure that the mapped data used for their study will correspond with their construct of visitation. Critically, the meaning of “exposure” for a particular study is relevant to how greenspace is delineated and interpreted. For example, should participants driving through parks or greenspace be considered as visitations, or only if participants walk through or stop within those areas? Should the mapped boundary of the park or greenspace be considered strictly, or should participants who are within the viewshed of the park or greenspace be considered exposed?

Parks serve multiple social and personal roles within communities, of which greenspace exposure is only one: indeed, some municipal parks do not include greenspace at all, including some of Philadelphia’s neighborhood playgrounds and playlots. Studies of greenspace typically do not provide a clear definition of this construct [44], which complicates the evaluation of relevant data sources, limits replicability, and could make it more difficult for policymakers to draw guidance from scientific literature. Deliberate framing of hypotheses connecting parks or greenspace with health outcomes, careful data selection, and willingness to edit existing data sources can build theoretical clarity and help to avoid issues of misclassification.

## Software

All mapping and spatial analysis was conducted using ArcGIS Pro version 3.1.2.

## Data Availability

No datasets were generated or analysed during the current study.
